# Plant plastids: from evolutionary origins to functional specialization and organelle interactions

**DOI:** 10.1093/jxb/eraf378

**Published:** 2025-08-20

**Authors:** Luciana Renna, Alessio Papini, Stefano Mancuso, Federica Brandizzi, Giovanni Stefano

**Affiliations:** Department of Agricultural, Food, Environmental and Forestry Sciences Technologies (DAGRI), University of Florence, Sesto Fiorentino, 50019 Firenze, Italy; Department of Biology, University of Florence, Florence 50121, Italy; Department of Agricultural, Food, Environmental and Forestry Sciences Technologies (DAGRI), University of Florence, Sesto Fiorentino, 50019 Firenze, Italy; Department of Plant Biology, Michigan State University, East Lansing, MI 48824, USA; MSU-DOE Plant Research Lab, Michigan State University, East Lansing, MI 48824, USA; Great Lakes Bioenergy Research Center, Michigan State University, East Lansing, Michigan, USA, MI 48824, USA; Department of Biology, University of Florence, Florence 50121, Italy; The University of North Carolina at Chapel Hill, USA

**Keywords:** Endomembrane compartment, endosymbiont, inter-organellar communication, membrane contact site, organelle, plastid, protein import

## Abstract

Plastids are highly diverse organelles that play critical roles in supporting many forms of life on Earth. Among them, chloroplasts house the machinery for photosynthesis, providing phototrophic capabilities to eukaryotes such as plants, algae, and photosynthetic protists. The functions of plastids are indispensable for the survival and development of life, and they are widely recognized as endosymbiotic organelles with a single origin. They exhibit morphological diversity, tissue specificity, and the ability to adapt to specific cellular functions. Despite this level of understanding, significant questions remain unanswered, such as how genetic material from the endosymbiont was transferred and integrated into the host nucleus, the timeline for the full integration of the endosymbiont into the host cell, and the processes by which plastids specialized and adapted to various cell types. While plastids have unique features and specialized roles, they are neither autonomous nor physically isolated. Instead, they interact with other sub-cellular compartments through yet-to-be-characterized membrane domains or specialized structures. This review explores the origin and evolution of plastids, their protein-import machinery, compartmentalization, and interactions with other cellular compartments, and highlights key unanswered questions in these areas.

## Teaser

The origins, genome evolution, protein-import machinery, and communication mechanisms of plastids within cells reveal how their internal organization and connections with other compartments support essential life processes beyond photosynthesis.

## Introduction

In higher plants, plastids represent a diverse group of organelles that share common features and play critical roles in various cellular processes, including energy production and lipid biosynthesis. Plastids exist in several forms, each with distinct characteristics that support different and specific developmental and metabolic processes at the organ, tissue, and cellular levels (Choi *et al.*, 2021). Notably, many plastids can change their identity through an interconversion process during development (Rolland *et al.*, 2018). This remarkable plasticity, which enables them to activate or deactivate specific functions depending on the tissue they occupy, gives plastids their name (Haeckel and Haeckel, 1866; Schimper, 1883; Sakamoto *et al.*, 2008). Although plastid interconversion has been well studied in land plants, its occurrence and regulation in algae remain less understood and might be more constrained due to differing cellular architectures and developmental programs.

The origin of plastids can be traced back to early photosynthetic eukaryotic cells, which emerged when a photosynthetic prokaryote, such as a cyanobacterium, was engulfed and subsequently became essential for autotrophic survival. Over time, this engulfed photosynthetic prokaryote evolved into a plastid, assuming key cellular functions (Chen *et al.*, 2018). This primary endosymbiotic event not only marked the origin of photosynthetic plastids but also laid the foundations for subsequent functional diversification among plastid-derived organelles. A crucial adaptation during the primary endosymbiotic process was the development of interactions with other cellular compartments. These interactions enabled the synchronization of plastid activities with the host cell and facilitated the exchange of biomolecules, establishing the foundation for a stable symbiotic relationship (Mueller-Schuessele and Michaud, 2018). While this symbiotic integration is shared across all primary plastid lineages, including glaucophytes, red algae, and green algae, the specific nature and complexity of inter-organellar communication has been most extensively characterized in *Embryophyta*. Over evolutionary time, these interactions became more sophisticated and stable, leading to the incorporation of most plastid genes into the nucleus of the host cell. Despite this genomic integration, plastids retained essential chloroplast genes necessary for their proper functioning and the sustenance of life. This host–organelle genomic coordination, involving the import of nucleus-encoded proteins, is a hallmark of all plastid-containing eukaryotes, although the components and regulation of import machinery can vary across lineages.

As the number of plastids per cell increased, ranging from one to as many as a hundred (Mache and Lerbs-Mache, 2001), the complexity of their integration within plant cells also grew. Coordination between plastids and other organelles became vital to maintain their collective behavior within a well-integrated system. Evidence suggests that plastids can form physical and functional associations with other organelles, such as mitochondria, peroxisomes, and the endoplasmic reticulum (ER), presumably to exchange metabolites and information (Hanson and Hines, 2018; Mathur *et al.*, 2022; Bian *et al.*, 2023; Wang *et al.*, 2023). These interactions can occur through transient associations or specialized structures such as membrane contact sites (MCSs), where the membranes of two organelles come into close proximity (∼10–80 nm) without fusing (Bian *et al.*, 2023). Although organelle interactions have also been observed in algal cells, the formation and functional roles of MCSs are less well characterized compared to terrestrial plants.

Within the cell, plastid metabolism is coordinated with broader cellular processes through various transporters located in the plastid envelope (Facchinelli and Weber, 2011). Additionally, tubular extensions of the stroma, the soluble matrix of the plastid, can serve as potential communication pathways with other cellular components (Hanson and Conklin, 2020). These tubules, termed stromules, are particularly prominent in land plants, where they have been implicated in signaling and metabolic exchange, although stromule-like structures have also been sporadically observed in some green algae (Gray *et al.*, 2001). Plastid–organelle interactions, including the formation of MCSs, are increasingly recognized as essential sites for nutrient exchange and metabolic regulation, supporting processes including lipid synthesis, photosynthesis, photorespiration, and plastid biogenesis (Jarvis and López-Juez, 2013; Mathur *et al.*, 2023). However, the extent to which these mechanisms operate in non-embryophyte plastid-containing lineages remains an open question.

This review mainly focuses on *Embryophyta*, while other lineages have been considered to explain the origin, evolution, protein-import machinery, compartmentation, and interactions of plastids with other plant cell compartments. Special attention is given to the emerging roles of plastid–organelle interactions and their importance in synchronizing cellular responses to environmental and metabolic signals (Bobik and Burch-Smith, 2015).

## Cellular origins, genome evolution, and protein-import machinery of plastids

**Fig. 1.**
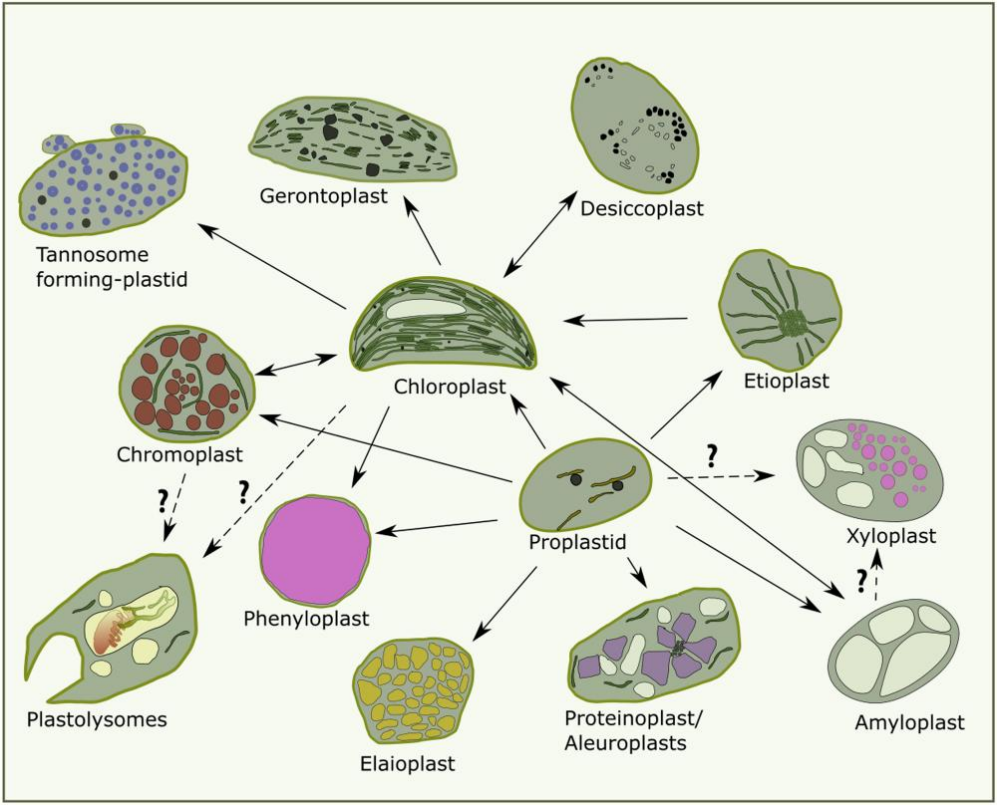
An overview of the different types of plastids that have been classified. The connecting arrows show how plastids differentiate, whilst the presence of question marks and dashed lines denotes potential routes that have not been thoroughly researched (see Box 1 for details).

Plastids are ubiquitous organelles present in all plants, including parasitic species (Ravin *et al.*, 2016). The origin of plastids, particularly chloroplasts, has long intrigued plant biologists (see Box 1 and Fig. 1 for an overview of plastid types). Over 140 years ago, Schimper first noted the similarities between plastids and cyanobacteria, hypothesizing that chloroplasts evolved from a symbiotic relationship between an early eukaryotic cell and a photosynthetic prokaryote (Schimper, 1883; Ward, 1883; Dyall *et al.*, 2004; Maréchal, 2018; Solymosi *et al.*, 2018). This idea was expanded by C. Mereschkowsky in the early 20th century, who proposed the groundbreaking theory of plastids as endosymbiotic organisms. Although initially dismissed, this theory gained widespread acceptance in the 1970s thanks in particular to the work of Margulis (Sagan, 1967; Margulis, 1970).

Box 1. **Plastid types and their known and unknown functions**

Plastids can be divided into green and non-green types, the latter being found exclusively in roots, seeds, tubers, and some fruits (Jarvis and López-Juez, 2013).

**Eoplasts or proplastids**

These are undifferentiated, non-green plastids that serve as the foundational forms from which all specialized plastid types (e.g. chloroplasts for photosynthesis and amyloplasts for starch storage) develop or re-differentiate. They have a spherical shape of ∼1 μm in diameter, contain ribosomes and plastoglobules, and have fewer inner membranes than etioplasts or chloroplasts (see Fig. 1). Proplastids are detected in developing meristems. Within them, the internal membrane architecture, specifically the thylakoid membrane system, is remarkably underdeveloped and far less abundant than the extensive thylakoid networks found in mature chloroplasts. It is characterized by the presence of only a limited number of unstacked tubules or nascent vesicles, which often maintain a direct structural continuity with the inner plastid membrane. Crucially, unlike the chlorophyll-rich thylakoids of chloroplasts, these internal membranes of proplastids do not usually contain chlorophyll, reflecting their non-photosynthetic and undifferentiated state. This contrasts sharply with the highly organized, interconnected network of chlorophyll-containing thylakoids and grana found freely within the stroma of mature, differentiated plastids such as chloroplasts. Two main types of proplastids have been identified: germinal and nodule (Solymosi *et al.*, 2018). Germinal proplastids have been discovered in meristems, dividing zones, reproductive tissues, and undifferentiated tissues (e.g. cell cultures and calli). Proplastids are metabolically active in developing tissue (Hedden, 2020). Nodule proplastids play a vital role in nitrogen fixation by metabolizing glutamine, which is derived from ammonia produced by bacteria after N_2_ reduction.

All other kinds of plastids originate from the proplastids, e.g. etioplasts (which develop in light-deprived photosynthetic tissues), chloroplasts (the photosynthetically active green plastids that are easily observed under a light microscope because of their pigmentation), chromoplasts (colored plastids that play a crucial role in ecological mechanisms such as attraction of pollinators or repulsion of herbivores), and leucoplasts (colorless plastids that are active in cellular functions such as storage and secretion) (Solymosi *et al.*, 2018).

Light deprivation or extremely low light conditions cause a developmental arrest of the proplastid-to-chloroplast transition. These transitionary plastids are called etioplasts and they contain prolamellar bodies. Etioplasts are characteristic of dark-grown shoots but not of other light-deprived tissues such as roots, and they have been used to study chloroplast development.

**Chloroplasts**

These are certainly the most-studied and best-described plastids, and are located mainly in leaf cells or green stems. Most of them are found in mesophyll cells, chlorenchyma tissues, guard cells, or embryo cells.

**Chromoplasts**

These are colored plastids containing red, yellow, or orange carotenoids. They can be found in fruits, flowers, leaves, and roots. The endomembranes of the chromoplasts lose their characteristic stromal and granular structure. Lipid droplets and fibrils, which are large supramolecular structures found in chromoplasts, have carotenoid-rich cores. The outer layer of the fibril structure is primarily a protein called fibrillin (Singh and McNellis, 2011; Nacir and Bréhélin, 2013; Llorente *et al.*, 2020). Chromoplasts are most often formed from chloroplasts, but they can revert. They can also develop from proplastids or leucoplasts. There are even examples of amyloplasts turning into chloroplasts and then into chromoplasts (Choi *et al.*, 2021).

**Leucoplasts**

These are colorless plastids that can differentiate from proplastids, chromoplasts, or chloroplasts. They are predominantly found in seed and root tissues, as well as other storage organs. Characterized by a limited inner membrane system and few thylakoids, the term ‘leucoplast’ encompasses several specialized subtypes, as follows.

*Amyloplasts.* These leucoplasts specialize in the synthesis and storage of starch; they typically contain large starch grains, which can reach diameters of up to 30 µm.

(Aloni *et al.*, 2004). In root columella cells, amyloplasts function as ‘statoliths’ and play a crucial role in gravitropic growth and responses (Aloni *et al.*, 2004). *Elaioplasts (or elioplasts).* These leucoplasts are adapted for the synthesis and storage of oils, and produce specific fatty acids

*Proteinoplasts (or proteoplasts/aleuroplasts).* These leucoplasts are involved in protein synthesis and storage. They accumulate large protein structures, often spherical or crystalloid-like, enclosed by a membrane (Papini and van Doorn, 2015).

It is important to note that leucoplasts, in general, lack the extensive endomembrane system characteristic of chloroplasts.

**Other plastids**

Other tissue- and stage-specific plastids have been characterized, raising new questions on the various plastids that can be present at different stages of plant development.

*Phenyloplasts.* These are chloroplasts that have lost their chlorophyll, giving the tissue a white color, and they have re-differentiated into phenol-containing plastids. In vanilla fruit, the phenyloplasts act as storage compartments for glucosylated phenolics (Brillouet *et al.*, 2014; Pinard and Mizrachi, 2018).

*Xyloplasts.* These have been found in secondary vascular tissues (xylem). These plastids mature from either amyloplasts or proplastids, and are where active synthesis of mono-lignols and biosynthesis of secondary cell wall precursors take place (Sundell *et al*., 2017; Pinard and Mizrachi, 2018).

*Gerontoplasts.* These develop from chloroplasts during senescence (Rolland *et al.*, 2018). It is believed that gerontoplasts regulate the breakdown of photosynthetic machinery and the membranes that host it through the formation and accumulation of plastoglobuli that contain the degradation products generated by the thylakoid membrane. These plastids are crucial for catabolism and remobilization processes of 75% of total leaf proteins. Amino acids and nitrogen from this process are used by the other tissues of the plant to grow. The chloroplasts located in the guard cells are the last to senesce because they control leaf gas exchange until the last moment. In gerontoplasts, thylakoids are stacked, and they do not disappear in a time-dependent way.

*Desiccoplasts (or xeroplasts).* These are atypical found in drought-resistant plants such as resurrection plants (Solymosi *et al.*, 2013; Lindquist *et al.*, 2016). During dehydration, chloroplasts decompose their chlorophylls and dismantle their thylakoid membrane system, becoming desiccoplasts: the conversion has been shown to be reversible once growth conditions are favorable again (Solymosi *et al.*, 2013).

*Tannosome-forming plastids.* These novel, plastid-derived structures were discovered relatively recently, and are endomembrane structures derived from chloroplasts after the thylakoids are disassembled. This process gives rise to the formation of pearl-like structures of ∼30 nm where tannin polymerization occurs. They are then encapsulated in a structure called a tannosome, which is generated by the fusion of both chloroplast envelopes (Pinard and Mizrachi, 2018). The tannosomes containing tannins are then shuttled towards the vacuole, where they are engulfed and stored.

*Plastolysomes (or autophagic-plastids).* Last but not least, these spherical or cup-shaped plastids have been described in some plants and appear to possess the ability to take up and hydrolyse cytoplasm that contains endoplasmic reticulum and other membranes. This type of autophagic activity has been documented only in some plant genera (Papini and van Doorn, 2015). The possibility of autophagy taking place in the plastid itself has been considered but has not yet been fully explored (Wan *et al.*, 2023).

**Fig. 2.**
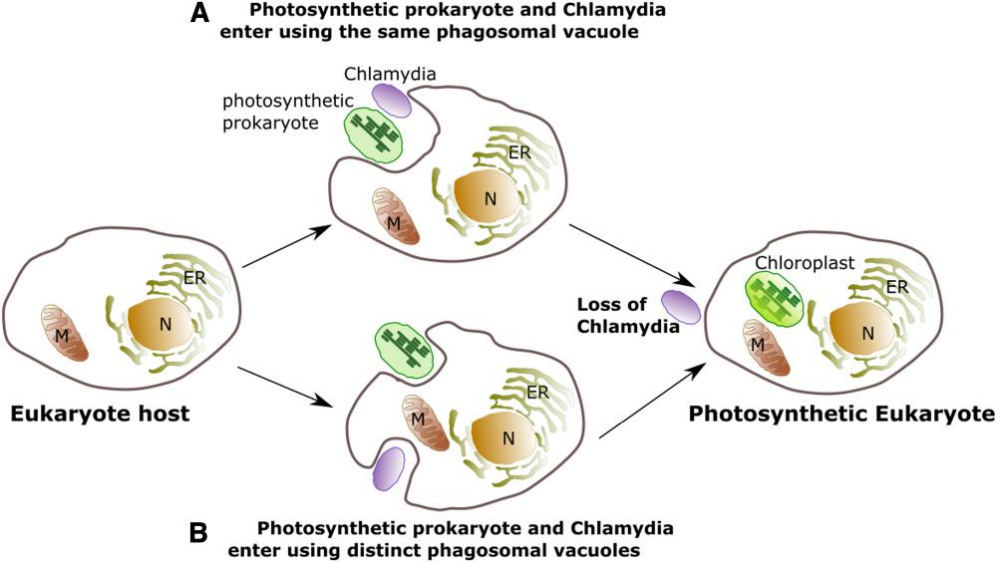
Representation of the ‘ménage à trois’ model of plastid genesis. The diagram shows a eukaryotic host encountering an ancestral cyanobacterium (a photosynthetic prokaryote) and *Chlamydia*. Both of these organisms entered the eukaryotic host cell simultaneously, either in the same phagosomal vacuole (A) or in separate phagosomal vacuoles (B). In both cases, *Chlamydia* was lost shortly after, giving rise to the photosynthetic eukaryote. ER, endoplasmic reticulum; M, mitochondrion; N, nucleus.

According to this endosymbiotic model, over a billion years ago a gram-negative cyanobacterium was engulfed by a eukaryotic host via phagocytosis, which then led to a symbiotic relationship (Fig. 2). The cyanobacterium subsequently evolved into an organelle capable of photosynthesis, converting sunlight into chemical energy and atmospheric CO_2_ into complex organic molecules. This newly formed organelle resulted in what we now identify as the chloroplast. Cytological and biochemical studies over the past five decades have provided strong evidence supporting this theory. Plastids share many features with cyanobacteria, including DNA sequence homology, mRNA translation machinery, and fatty acid biosynthesis pathways (Martin *et al.*, 2002; Ryall *et al.*, 2003; Tiller and Bock, 2014). The presence of transmembrane-barrel proteins (β-barrel) in the chloroplast outer envelope, resembling those of cyanobacteria, further reinforces their shared ancestry (Baers *et al.*, 2019).

Recent genome sequencing of photosynthetic eukaryotes has unveiled a fascinating twist. Up to 55 plastid-related genes appear to have a Chlamydial origin, often containing plastid-targeting signals (Brinkman *et al.*, 2002; Moustafa *et al.*, 2008). This suggests that *Chlamydia*, an obligate intracellular bacterium, may have coexisted with cyanobacteria during the early stages of plastid evolution. This idea forms the basis of the ‘ménage à trois hypothesis’ (MATH), which proposes that a tripartite interaction between a eukaryotic host, a cyanobacterial endosymbiont, and a *Chlamydiales* bacterium played a pivotal role in plastid genesis (Ball *et al.*, 2013; Facchinelli *et al.*, 2013a; Cenci *et al.*, 2017; Maréchal, 2018; Fig. 2). According to this model, *Chlamydiales* might have contributed genes that facilitated the establishment and integration of the endosymbiotic cyanobacterium by providing key functions such as metabolite transport and protein trafficking. However, the hypothesis remains controversial, primarily due to the weak and often ambiguous phylogenetic signals that support a Chlamydial origin for these genes. Critics argue that such signals might arise from methodological artifacts or unrelated horizontal gene transfers, leading to ongoing debate over the validity of the model. Therefore, while MATH offers a compelling framework for understanding the complex origins of plastids, further investigation is needed to clarify the evolutionary contributions of *Chlamydiales* and to resolve the phylogenetic uncertainties surrounding this hypothesis. Through evolutionary processes, the symbiotic relationship between the host and the cyanobacterium became stable, allowing for extensive gene transfer from the endosymbiont to the host genome (Rousseau-Gueutin *et al.*, 2016). While the precise mechanisms of this transfer remain unclear, evidence suggests that plastids retained a reduced genome, or plastome, containing ∼120 genes coding for ribosomal components, tRNAs, and organellar proteins. This streamlined genome, often circular or linear (Bendich, 2004), is insufficient for autonomous function, requiring significant interaction with the host for processes such as division, membrane biogenesis, and metabolism.

**Fig. 3.**
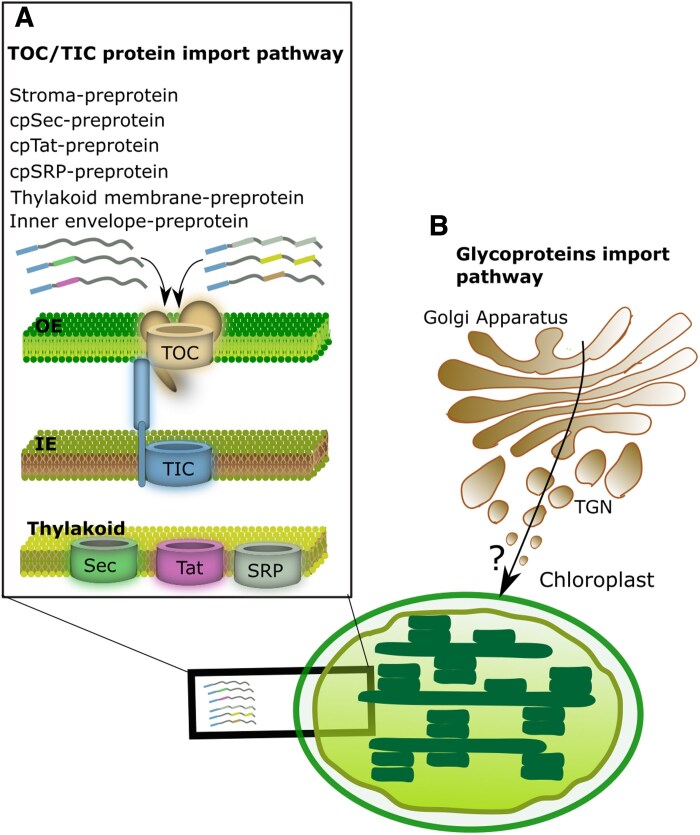
Overview of protein import in plastids. (A) The Translocon at the Outer Chloroplast Membrane/Translocon at the Inner Chloroplast Membrane (TOC–TIC) complex is localized on the chloroplast membrane, with TOC present on the outer envelope (OE) and TIC on the inner envelope (IE; together, they are responsible for the import of the majority of chloroplast preproteins. Proteins in the stroma containing luminal-targeting peptides are mostly sorted via the chloroplast secretory (cpSec) and chloroplast twin-arginine translocation (cpTat) pathways, whereas thylakoid-membrane proteins are predominantly targeted through the chloroplast signal recognition particle (cpSRP) pathway and by spontaneous insertion. (B) Glycosylated proteins appear to enter the chloroplast through the Golgi apparatus by means of an unidentified translocation or import machinery. TGN, trans-Golgi network as TGN.

The integration of plastids into the host cell involved significant adaptations, including the elimination of the bacterial cell wall and the development of a double-membrane envelope. Glaucophyte plastids (muroplasts) stand out as an exception, retaining a remnant of the ancestral cyanobacterial cell wall. This primitive characteristic positions glaucophytes as a key reference point for reconstructing early stages of plastid evolution (Facchinelli *et al.*, 2013b). Consistent with their more ancestral state, glaucophyte plastids also appear to possess fewer host-derived, nuclear-encoded transporters than the more evolutionarily advanced plastids of red and green algae (Facchinelli *et al.*, 2013b). These observations support the hypothesis that the loss of the bacterial cell wall was a crucial precondition for the effective acquisition and integration of host transport systems—an essential step in the transition from endosymbiotic cyanobacteria to fully integrated plastids. (Rousseau-Gueutin *et al.*, 2016). Another critical adaptation was the evolution of protein-import machinery. Over 95% of chloroplast proteins are now encoded in the host nucleus, translated in the cytosol, and imported into the plastid via translocon complexes (Richardson and Schnell, 2020). The translocon at the outer chloroplast membrane (TOC) and translocon at the inner chloroplast membrane (TIC) complexes facilitate this process (Fig. 3A).

The TOC complex mediates initial protein import. Its key components include Toc159 and Toc34, receptor proteins that recognize transit peptides (TPs), and Toc75, a channel-forming protein that translocates proteins across the outer membrane (Richardson *et al.*, 2014). Once inside the intermembrane space, the TIC complex facilitates protein transfer across the inner membrane. Major TIC components include Tic110, a protein whose function is still debated but that is thought to work as a scaffold recruiting stromal chaperones (Inaba *et al.*, 2005; Kovacheva *et al.*, 2005; Tsai *et al.*, 2013); Tic40, which stabilizes interactions; Tic20, a channel protein; and others such as Tic56, Tic100, Tic214, and Tic236, which contribute to structural integrity and import efficiency (Schnell, 2018; Richardson and Schnell, 2020; Jin *et al.*, 2022; Liu *et al.*, 2023; Sun and Jarvis, 2023).

After crossing both membranes, proteins reach the stroma, where stromal processing peptidase cleaves their TPs, enabling proper folding and function (Richter and Lamppa, 1999; Jarvis and Kessler, 2014). Some proteins are further directed to the thylakoid lumen and membranes, the site of photosynthesis, with lumenal proteins mainly sorted via the chloroplast secretory (cpSec) and chloroplast twin-arginine translocation (cpTat) pathways (Nellaepalli *et al.*, 2023). In contrast, thylakoid membrane proteins are predominantly targeted through the chloroplast signal recognition particle (cpSRP) pathway or by spontaneous insertion (Fig. 3) (Jarvis and Robinson, 2004; Xu *et al.*, 2021). Evidence also suggests that glycosylated proteins synthesized in the ER can reach plastids via vesicular transport or MCSs, although the precise mechanisms remain unclear (Villarejo *et al.*, 2005; Kaneko *et al.*, 2016).

The coordinated action of TOC and TIC complexes demonstrates the co-evolution of these systems, ensuring efficient protein import and plastid integration within the cellular network (Richardson and Schnell, 2019). This intricate machinery supports the high demand for chloroplast proteins during critical growth stages, such as seedling development, and underscores the importance of plastid–nucleus communication in sustaining plant life (Bölter and Soll, 2016).

## Plastid specialization and structure

Plastid compartmentation and function are highly species- and cell-type-specific. Beyond their role in light-driven carbon fixation (in the case of chloroplasts), plastids play essential roles in numerous processes, including photorespiration, chlorophyll synthesis, oxygen metabolism, fatty acid and lipid synthesis, and in the biosynthesis of vitamins (E and K), pigments, amino acids, and hormones (Bowsher and Tobin, 2001; Rolland *et al.*, 2018). Plastids are also critical for starch synthesis and other key metabolic pathways, serving as hubs for essential biochemical processes (Hwang *et al.*, 2020). In the green lineage, starch synthesis occurs specifically within the plastids, whereas in *Rhodophyta* and *Glaucophyta*, starch is synthesized outside the plastids (Viola *et al.*, 2001; Deschamps *et al.*, 2008). Additionally, they possess the machinery necessary to replicate their own genome and reduce nitrite and sulfate.

Our current understanding of plastid diversity is reshaping our view of how organelles evolve and how metabolic functions diversify in eukaryotes.

While the primary acquisition of photosynthetic organelles was exceedingly rare, the recurrent establishment of tightly integrated non-photosynthetic bacterial endosymbionts across eukaryotic supergroups highlights a pervasive selective pressure for novel metabolic capacities, notably nitrogen fixation. The recently characterized nitroplast of *Epithemia clementina* and the related UCYN-A symbiont of *Braarudosphaera bigelowii* exemplify a remarkable evolutionary trajectory toward organellogenesis from such nitrogen-fixing cyanobacterial endosymbionts. These ‘spheroid bodies’ as Nowack and Weber (2018) described them, derived from the same cyanobacterial lineage, demonstrate that nitrogen fixation, a traditionally prokaryotic function, has become fully integrated into eukaryotic cell architecture as a heritable organelle. This profound process involves significant host–symbiont co-evolution, evidenced by extensive genome reduction in the symbiont, synchronized division with the host, and the essential import of host-encoded proteins that complement the metabolic pathways of the organelle. These newly characterized organelles exhibit significant genome reduction, often including the loss of oxygenic photosynthesis, and an intimate metabolic integration with their hosts, necessitating hypothesized host-derived metabolite exchange. Although some questions persist regarding the precise mechanisms of host-protein import across the encompassing host-derived membrane and the full extent of genetic integration, these nitroplast systems represent compelling models for studying the ongoing evolution of nitrogen-fixing organelles, underscoring the remarkable plasticity of endosymbiotic evolution and suggesting that such genetic integration is indeed achievable over evolutionary time (Nowack and Weber, 2018; Coale *et al.*, 2024; Moulin *et al.*, 2024).

Plastid organization differs significantly between C_3_ and C_4_ plants due to their distinct modes of photosynthesis. In C_3_ plants, photosynthesis occurs entirely within mesophyll cells, where chloroplasts are relatively uniform in both structure and function. These chloroplasts contain well-developed grana, which support the full complement of light-dependent reactions, including both PSI and PSII. The chloroplasts are distributed throughout the cytoplasm of mesophyll cells and are primarily responsible for fixing atmospheric CO_2_ through the Calvin cycle.

In contrast, C_4_ plants exhibit a specialized leaf structure known as Kranz anatomy, which is characterized by a spatial separation of the initial carbon fixation and the Calvin cycle between two distinct cell types: mesophyll cells and bundle sheath cells. In mesophyll cells of C_4_₄ plants, chloroplasts are typically rich in grana and are involved in the initial fixation of CO_2_ into four-carbon compounds via phosphoenolpyruvate (PEP) carboxylase. These compounds are then transported to the bundle sheath cells, where chloroplasts differ markedly in structure and function. Bundle-sheath chloroplasts usually lack or have only rudimentary grana and are specialized for the Calvin cycle, relying primarily on PSI. Additionally, these chloroplasts are often arranged closer to the vascular tissue and frequently accumulate starch because of their central role in carbon fixation. Thus, the compartmentalization of plastid function in C_4_ plants enables a more efficient photosynthetic process under high light and temperature conditions, in contrast to the more uniform plastid organization seen in C_3_ plants (Lascève *et al.*, 1997; Wise, 2007).

While plastids are known to contain sub-compartments such as the envelope, stroma, and thylakoid membranes, some lack well-developed thylakoid membranes, which together support their diverse metabolic functions. However, not all plastids exhibit fully developed thylakoid systems—particularly in non-photosynthetic or certain developmental stages. In addition to these major compartments, plastids also contain specialized structures including plastoglobules, which are lipid-rich bodies involved in lipid metabolism and stress responses; prolamellar bodies (PLBs), which appear in etioplasts and facilitate the transition to photosynthetic activity upon light exposure; and the peripheral reticulum (PR), an extension of the inner-envelope membrane thought to enhance metabolite exchange. Together, these sub-structures reflect the functional and developmental versatility of plastids across different cell types and environmental conditions (Austin *et al.*, 2006; Charuvi *et al.*, 2012; Cortleven *et al.*, 2016).

### The plastid envelope

The plastid envelope, a double-membrane system comprising inner and outer membranes, regulates communication with the cytosol and neighboring membranes. It acts as a selective barrier, mediating the exchange of proteins, lipids, and metabolites. In addition to its transport function, the envelope is critical for solute trafficking, pigment biosynthesis, and lipid metabolism—key processes that sustain plastid and cellular functions (Facchinelli and Weber, 2011; Wang and Benning, 2012). A distinguishing feature of the outer envelope membrane is the presence of β-barrel proteins—structurally reminiscent of those found in Gram-negative bacteria—which facilitate metabolite transport and highlight the evolutionary origin of plastids from cyanobacterial endosymbionts. The envelope also houses the TOC–TIC translocon complex (Schnell, 2018; Richardson and Schnell, 2020; Fig. 3) and the CHLORAD degradation system, a cytosolic ubiquitin-proteasome-dependent pathway essential for organelle function and plant growth (Ling *et al.*, 2019; Sun *et al.*, 2022). The space between the outer and inner membranes contains enzymes critical for pigment and lipid metabolism, further contributing to cellular homeostasis and plastid functionality (Cook *et al.*, 2021).

The envelope also generates stroma-filled tubular extensions that facilitate interactions with other organelles (Kwok and Hanson, 2004a; Gray *et al.*, 2012). While the morphology of stromules is well described, their functional role, if any, remains unclear.

### The stroma

The stroma, a dense matrix abundant in proteins and enclosed by the inner-envelope membrane, is the sub-organellar environment for several metabolic processes, including carbon fixation and synthesis of amino acids, nucleotides, and lipids (Jensen *et al*., 2017). The thylakoids, membrane structures arranged in stacks known as grana and linked by stromal lamellae, are inside the stroma and contain the photosynthetic apparatus. The stroma contains the plastome, organized into multiple nucleoids (up to several hundred copies), along with transcription and translation machinery, including 70S ribosomes, ribosomal proteins, RNA polymerase, mRNA, and tRNAs (Bieri *et al.*, 2017; Greiner *et al.*, 2020).

### Thylakoid membranes

The thylakoid membranes form a highly organized internal network comprising cylindrical stacks called grana, which interconnect unstacked lamellae. These membranes enclose the thylakoid lumen, an aqueous matrix that plays key roles in critical processes such as oxygenic photosynthesis, water splitting, electron transport, chemiosmosis, and ATP synthesis (Staehelin and Paolillo, 2020). The photosynthetic electron transport chain, including PSI and PSII, is embedded within these membranes, with PSII densely packed in the grana and PSI located in unstacked stromal thylakoids (Kirchhoff *et al.*, 2007; Gu *et al.*, 2022).

The sub-compartmentation of the thylakoid membranes supports diverse biosynthetic pathways, such as amino acid and fatty acid biosynthesis, nitrogen and sulfur assimilation, and pigment and hormone accumulation (Peltier *et al.*, 2000; Joyard *et al.*, 2010). Embedded within or associated with these membranes are plastoglobules (PGs), which are lipid–protein particles whose abundance and function vary with plastid type and developmental stage. PGs are dynamic structures involved in tocopherol biosynthesis, jasmonate metabolism, and antioxidant distribution. They also store excess lipids such as plastoquinone and α-tocopherol, regulate redox and photosynthetic reactions, and recycle thylakoid components during senescence or stress (Lichtenthaler, 2013; Van Wijk and Kessler, 2017). Structures that can be considered as thylakoid progenitors are the PLBs, which are unique to angiosperm plastids, are formed in darkness, and consist of tetrahedrally arranged, branched tubules containing protochlorophyllide and carotenoids (Gunning, 2001). Upon exposure to light, PLBs disassemble and give rise to organized thylakoid membranes during chloroplast differentiation, but their precise functions remain underexplored (Park *et al.*, 2002).

### Peripheral reticulum

The PR is a system of tubules and vesicles connected to the inner-envelope membrane and classified into three types (I–III) based on morphology. Type I consists of single or double rows of tubules situated beneath the inner-envelope membrane at the chloroplast periphery. Type II is characterized by dense aggregates of vesicles and tubules localized to specific regions of the plastid periphery, while Type III comprises individual vesicles budding directly from the inner-envelope membrane (Lindquist *et al.*, 2016). PR structures often increase in size under biotic or abiotic stress and might play a role in metabolite transport or stress responses, although their precise function remains speculative (Szczepanik and Sowiński, 2014).

## Plastid interactions

**Fig. 4.**
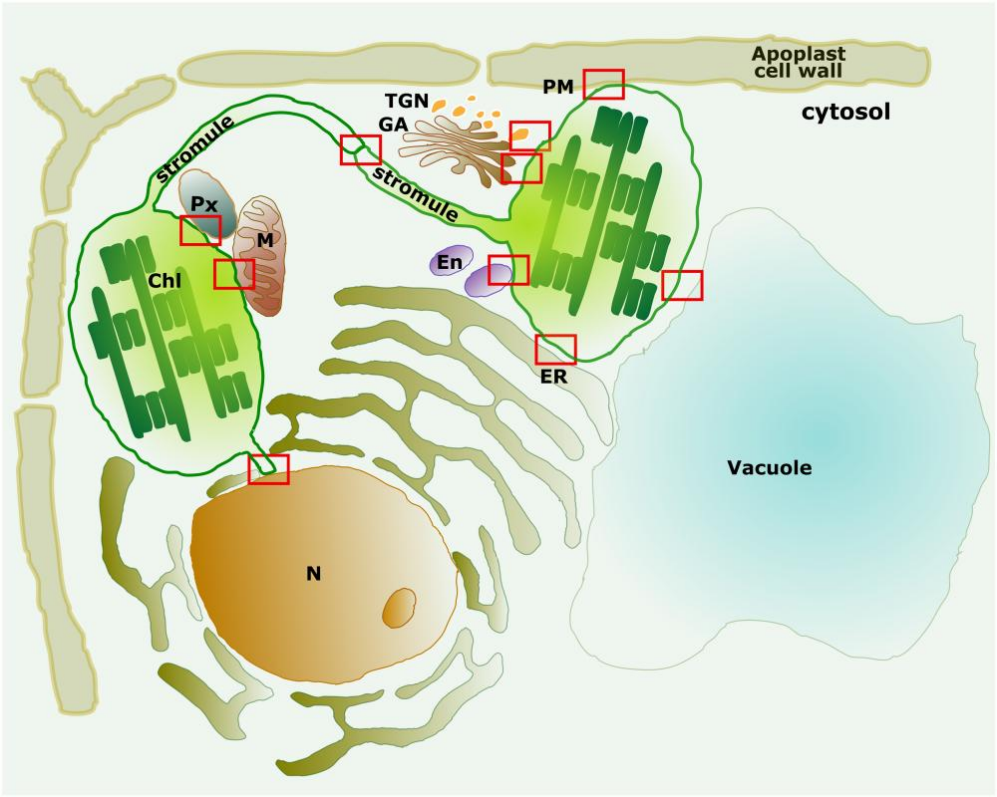
Schematic representation of the endomembrane system showing the close associations between plastids and other intracellular compartments. The diagram shows the spatial organization and the close relationships (rectangles) that have been reported so far between plastids (chloroplasts), endomembrane compartments, and other organelles within a plant cell. For some of these sites the exchange of proteins or biomolecules has not yet been demonstrated. Chl, chloroplast; En, endosome; ER, endoplasmic reticulum; GA, Golgi apparatus; M, mitochondrion; N, nucleus; PM, plasma membrane; Px, peroxisome; TGN, trans-Golgi network.

**Fig. 5.**
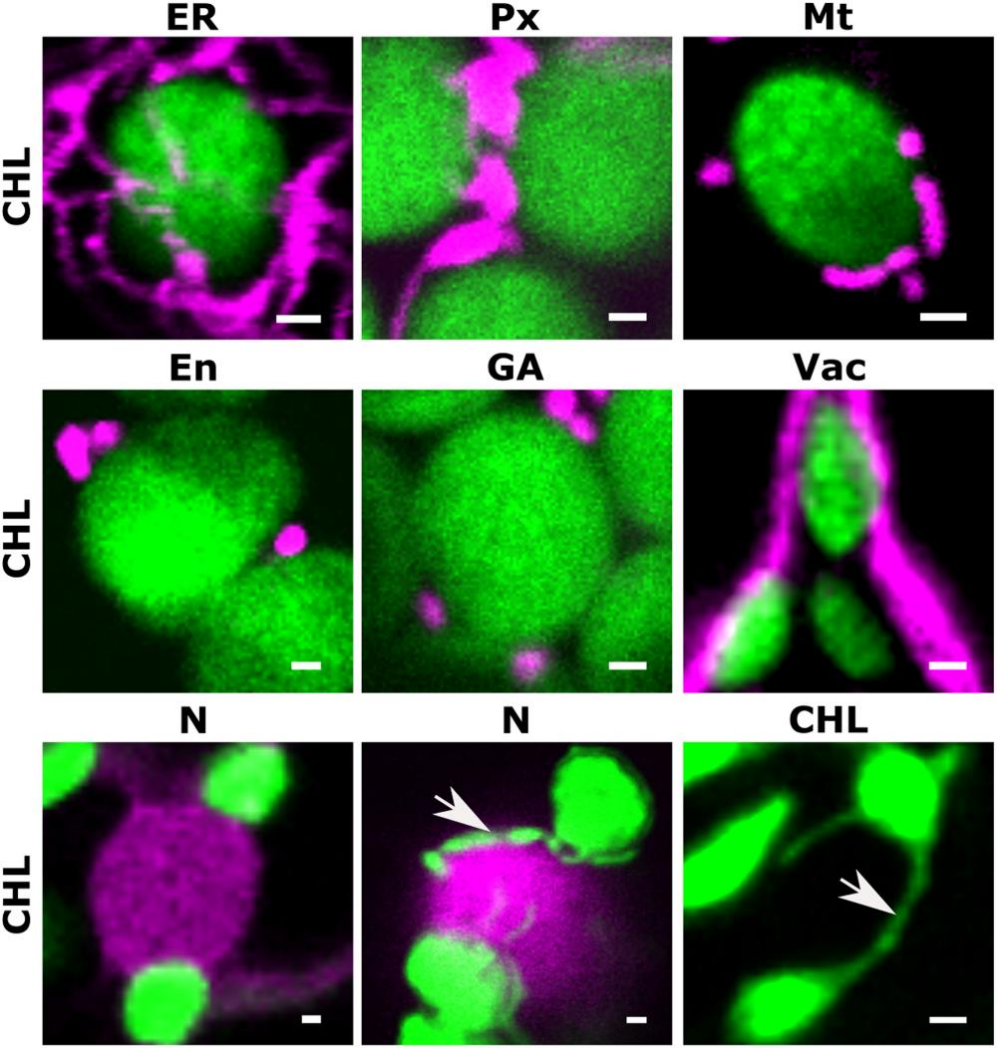
Confocal micrographs of Arabidopsis epidermal cells, providing illustrative examples of plastids (chlorophyll autofluorescence) and their spatial relationship with the endoplasmic reticulum (ER), peroxisomes (PX), a mitochondrion (MT), endosomes (En), the Golgi apparatus (GA), the vacuole (VAC), the nucleus (N), and chloroplasts (CHL). The plastids are sited in close proximity to the other organelles in the cell (magenta). The arrows indicate stromules. Scale bars are 1μm. These images serve to illustrate the various findings described in the following papers: Gaffal *et al.* (2007); Kitajima *et al.* (2009); Breuers *et al.* (2012); Mehrshahi *et al.* (2013); Caplan *et al.* (2015); Gao *et al.* (2016); Mullineaux *et al.* (2020); Wu *et al.* (2020); Oikawa *et al.* (2021); Mathur *et al.* (2023); and Renna *et al.* (2024).

**Fig. 6.**
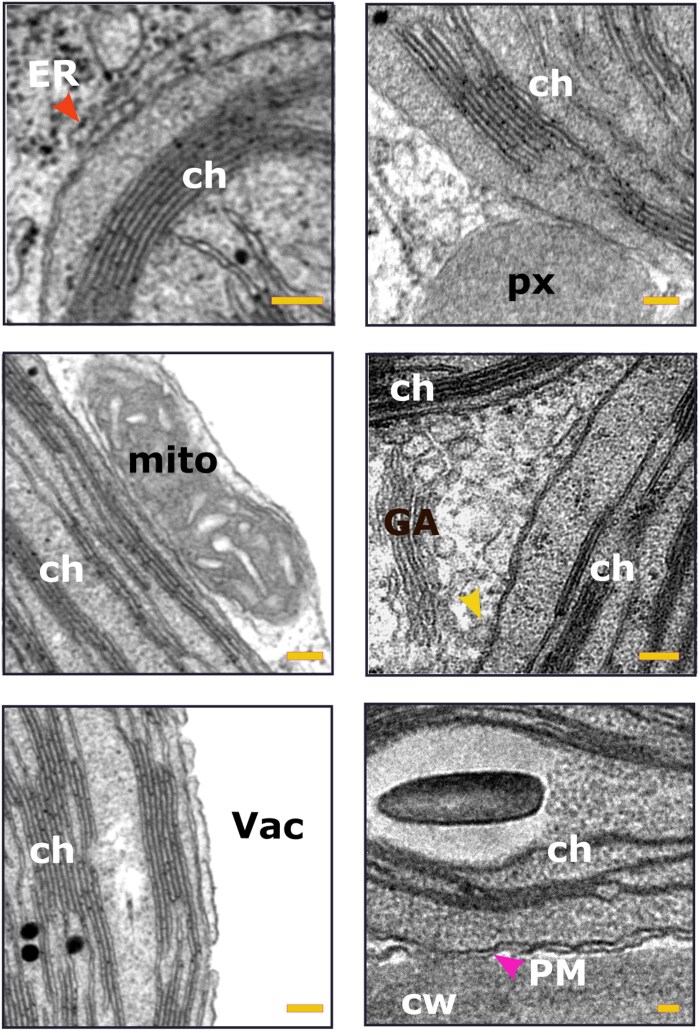
TEM micrographs of Arabidopsis providing illustrative examples of plastids positioned in close association with other organelles: ch, chloroplast; cw, cell wall; ER, endoplasmic reticulum; GA, Golgi apparatus; mito, mitochondrion; PM, plasma membrane; px, peroxisome; Vac, vacuole. The red arrowhead points to an ER tubule; the yellow arrowhead indicates a portion of the GA that is closely associated with the chloroplast outer envelope; and the magenta arrowhead indicates plasma membrane that is adjacent to the chloroplast. Scale bars are 100 nm. These images serve to illustrate the various findings described in the following papers: Gaffal *et al.* (2007); Oikawa *et al.* (2008, 2021); Kitajima *et al.* (2009); Breuers *et al.* (2012); Mehrshahi *et al.* (2013); Caplan *et al.* (2015); Gao *et al.* (2016); Mullineaux *et al.* (2020); Wu *et al.* (2020); Mathur *et al.* (2023); and Renna *et al.* (2024).

Recent advances in electron and fluorescence microscopy, along with improved imaging techniques, have provided unprecedented insights into the ultrastructure of plastids and their interactions with other organelles. These findings have transformed the traditional view of plastids as isolated units into a more dynamic concept of plastids connected to the endomembrane system (Barton *et al.*, 2018) (see Figs 4–6, and Table 1). Plastids are now recognized as highly dynamic organelles capable of reshaping their structure, composition, and positioning in response to developmental and environmental signals. Coordinating closely with other cellular compartments, plastids form specialized association sites whose structural presence is increasingly well documented and that are thought to facilitate the direct exchange of biomolecules (Mueller-Schuessele and Michaud, 2018; Mathur *et al.*, 2023; Huercano *et al.*, 2025), although the functional roles of these contact sites require further experimental validations. These interactions are proposed to enable plastids to integrate their metabolism with other organelles, optimizing responses to biotic and abiotic challenges and supporting plant growth and development.

**Table 1.** Putative proteins and functions of the close-association sites between plastids and organelles\endomembrane compartments

Plastid close-association site		Key proteins identified	Putative function	References
Chloroplasts	Endoplasmic reticulum	TGD1–5; LACS4 and LACS9; P4-ATPase family, Ala10, ALIS5; BnCLIP1; Vap27-ORP2A	Lipid trafficking	Breuers *et al.* (2012); Wang *et al.* (2012); Poulsen *et al.* (2015); Botella *et al.* (2016); Mathur *et al.* (2023); Renna *et al.* (2024)
Chloroplasts/plastids	Peroxisome	Pex10 (zinc RING finger domain)	Photorespiration	Schumann *et al.* (2007)
Chloroplasts/plastids	Mitochondria	MTL complex (AtMic60; OEP7-At3g52420; OMP24-At3g52230)	Energy metabolism; lipid trafficking	Michaud *et al.* (2016); van der Laan *et al.* (2016); Michaud (2018)
Chloroplasts/plastids	Plasma membrane	CHUP1	Lipid trafficking, positioning	Oikawa *et al.* (2008)
Chloroplasts/plastids	Endosomes	Unknown	Lipid trafficking? Iron trafficking?	–
Plastids	Vacuole	Unknown	Exchange, storage, and mobilization	Pacini *et al.* (2003)
Chloroplasts/plastids	Golgi apparatus and trans-Golgi network	NPP 1, 2, and 6	Molecule exchange	Nanjo *et al.* (2006); Kaneko *et al.* (2016)
Chloroplasts/plastids	Plastids	Unknown	Signalling?	–
Chloroplasts/plastids	Nucleus	Unknown	Anterograde and retrograde signalling	Kwok and Hanson (2004b); Mueller-Schuessele and Michaud (2018); Hanson and Conklin (2020)

Plastids serve as communication hubs for various cellular activities, from RNA processing and protein translocation to the regulation of enzymatic pathways (Ferro *et al.*, 2003; Barkan and Small, 2014; Paila *et al.*, 2015). This functional integration plays a key role in coordinating plant growth, reproduction, and responses to environmental stimuli (Muñoz and Munné-Bosch, 2020). Evidence suggests that plastids communicate with other organelles by releasing small signaling molecules, often referred to as ‘plastidic signals’ (Schattat *et al.*, 2012b; Woodson and Chory, 2012; Barton *et al.*, 2013; Mathur *et al.*, 2013; Hanson and Hines, 2018). These signals can trigger changes in gene expression and other cellular processes in target organelles, such as the nucleus (Osteryoung and Pyke, 2014; Mueller-Schuessele and Michaud, 2018). While the existence of plastid-derived signals is well supported, the precise molecular identities and mechanisms of action of these signals remain an active area of investigation. This ability to transmit signals across cellular compartments elevates plastids from passive metabolic units to active regulators of cellular behavior.

One of the most critical interactions for plastids is with the nucleus, given that plastids transferred most of their genetic material to the nuclear genome during evolution. This relationship is modulated by various factors, including light exposure, biotic and abiotic stresses, and developmental stages (Larkin, 2014; Mueller-Schuessele and Michaud, 2018; Mullineaux *et al.*, 2020). The evolution of this communication system was essential as plants transitioned from a single plastid per cell, typical in algae, to the multiple plastids found in most plant cells today. Synchronizing multiple plastids within a cell probably required the development of highly specialized signaling and interaction mechanisms. This reflects the evolutionary refinement of plastid–nuclear coordination, where plastids act not just as energy converters but as integrated controllers of cellular fate. Nonetheless, a mechanistic understanding of how such coordination is achieved at the molecular level remains limited.

Close associations among plastids within the same cell are influenced by plastid type and environmental conditions, such as stress (Breuers *et al.*, 2012; Hanson and Hines, 2018; Hanson and Conklin, 2020). Plastids also form heterotypic connections with other sub-cellular compartments, such as mitochondria, peroxisomes, and the ER. These interactions often involve MCSs, which are hypothesized to facilitate the direct transfer of metabolites and signaling molecules (Mueller-Schuessele and Michaud, 2018). However, the molecular machinery mediating these interactions remains largely unknown and is likely to be highly specific to each organelle pairing. Additional molecular and functional studies will be essential to validate the physiological significance of these contact sites and clarify their roles in cellular homeostasis.

In summary, plastids are not static metabolic units but dynamic, interactive organelles that function as nodes in a broader cellular network. These connections are essential for synchronizing plastid activity with the broader cellular environment, enabling plants to adapt and thrive under varying conditions (Dopp *et al.*, 2021; Altamura *et al.*, 2024; Kunjumon *et al.*, 2024).

### Plastid–nucleus interactions

Plastids continuously transmit signals to the nucleus to regulate their physiological status in response to environmental changes (Block and Jouhet, 2015). The mechanisms ensuring that these retrograde signals reach the nucleus intact and convey their full information remain poorly understood. Signaling molecules must traverse the chloroplast lipid bilayer and the cytosol while avoiding interference from other organelles to deliver accurate information to the nucleus. Proposed mechanisms include ‘chaperone proteins’ that facilitate active signal transport via the cytosol (Leister, 2012) and the formation of MCSs that act as direct communication channels between plastids and the nucleus (Mullineaux *et al.*, 2020).

Close associations between plastids and the nucleus, often accompanied by specialized plastid structures called stromules, have been observed (Kwok and Hanson, 2004b; Hanson and Conklin, 2020). Stromules are stroma-filled tubules projecting from plastid envelopes, frequently connecting to the nucleus (Köhler *et al.*, 1997; Köhler and Hanson, 2000; Caplan *et al.*, 2008, 2015; Mueller-Schuessele and Michaud, 2018). Studies in Arabidopsis have revealed that most stromules are oriented toward the nucleus, suggesting the existence of a ‘stromule-promoting zone’ where their formation is concentrated (Erickson *et al.*, 2017; Erickson and Schattat, 2018).

Stromules, akin to bacterial pili, might facilitate the transport of a wide range of signaling molecules, including proteins (Köhler *et al.*, 1997; Hanson and Conklin, 2020). Fluorescently tagged proteins localized to stromules have enabled real-time visualization of their formation under varying biotic and abiotic stress conditions (Schattat and Klosgen, 2011; Mathur *et al.*, 2012). Stromule emergence is influenced by growth conditions that enhance lipid synthesis and trafficking, with MCSs playing a critical role in their formation (Block and Jouhet, 2015). Under stress, stromules probably help maintain cellular homeostasis by facilitating well-coordinated communication between plastids and the nucleus (Gray *et al.*, 2012).

In a groundbreaking study, Köhler *et al.* (2000) demonstrated active, energy-driven transport within stromules, challenging the earlier assumption that they functioned only through passive diffusion. Using fluorescence correlation spectroscopy, the study showed that green fluorescent protein (GFP)-tagged molecules moved more slowly within stromules than in the cytosol, indicating active transport mechanisms. This finding suggests that stromules enable long-distance distribution of proteins and ensure their even distribution throughout plastid tubules. Future research is required to uncover the exact mechanisms of this active transport and its role in plastid function.

Stromule formation is induced by bacterial infections and pro-defense signals such as hydrogen peroxide (H_2_O_2_) and salicylic acid (Caplan *et al.*, 2015). During pathogen attack, stromule numbers and their connections to the nucleus increase, potentially facilitating the delivery of immune signals from chloroplasts to the nucleus and enhancing programmed cell death, a key component of plant immunity (Caplan *et al.*, 2015; Savage *et al.*, 2021). For example, during infection by *Phytophthora infestans*, chloroplasts form stromules, accumulate at the pathogen interface, and associate closely with the nucleus in infected cells (Savage *et al.*, 2021). This process is mediated by the immune signaling protein BAK1, and stromule formation is suppressed when BAK1 signaling is inhibited, highlighting its role in defense responses.

These observations suggest that stromules are not merely by-products of effector-triggered immunity but play active roles in signaling cascades, particularly in the polarized response to pathogen attack (Caplan *et al.*, 2015). However, stromules are not required for guiding plastid migration toward the nucleus (Kumar *et al.*, 2018; Prautsch *et al.*, 2023), emphasizing the need for further research into the mechanisms driving plastid–nucleus aggregation.

The exact mechanisms underlying the formation of stromules, their role in plastid–nucleus communication, and their involvement in plant immunity remain areas of active investigation. Stromules might serve as a critical avenue for rapid and efficient signaling during stress responses, positioning them as a promising target for future research into plant defense mechanisms. Unraveling these dynamic processes will deepen our understanding of organelle interplay and plant immune responses, opening new possibilities for improving crop resilience.

### Plastid–plastid interactions

The close associations between plastids and the role of stromules in these interactions remain underexplored. Early experiments using stromal-soluble GFP demonstrated the active movement of tagged proteins within stromules (Köhler *et al.*, 2000; Mechela *et al.*, 2019). Stromules have also been observed connecting different plastids (Kwok and Hanson, 2004a; Holzinger *et al.*, 2008; Breuers *et al.*, 2012). However, the longstanding belief that plastids are interconnected by a functional network of stromules has been challenged. Studies have shown that plastids labeled with distinct fluorescent proteins do not exchange these markers, even when connected by stromules (Schattat *et al*., 2012a, 2012b). Furthermore, it has been argued that plastids linked by stromules are semantically and functionally the same plastid (Schattat *et al*., 2012a, 2012b, 2015). These findings and opinions raise important questions about the actual function of stromules and their role in plastid–plastid interactions.

Further research using photoconvertible fluorescent proteins (pcFPs) has added complexity to this picture. These studies have demonstrated that pcFPs can transfer from a photoconverted plastid to another via a stromule (Breuers *et al.*, 2012; Hanson and Hines, 2018). However, it remains unclear whether plastids connected by stromules are functionally linked or represent progeny plastids that have not fully separated despite physical distance. This aspect of plastid interaction requires further investigation to reach definitive conclusions.

It has been hypothesized that stromules might facilitate the transport of ions or small molecules, or play a role in inter-organelle signaling (Schattat *et al.*, 2012a, 2015; Mathur *et al.*, 2013; Hanson and Hines, 2018). One speculative hypothesis suggests that stromules might act as components of signal transduction pathways, establishing direct connections between distant plastids. In this scenario, a signal originating in one plastid could propagate to adjacent plastids in a ‘chain reaction’ mechanism, enabling a coordinated cellular response. This concept bears some resemblance to bacterial quorum sensing, where diffusible signaling molecules allow bacteria to monitor and adjust cell density, enabling intercellular communication (Ram and Lo, 2018). Interestingly, many algae and non-vascular plants typically have a single plastid per cell, whereas vascular plants often contain multiple plastids. This difference suggests that stromules might represent an evolutionary innovation associated with the emergence of multiple plastids per cell (Natesan *et al.*, 2005; Hanson and Sattarzadeh, 2011; Miyagishima, 2011; Erickson *et al.*, 2023). Therefore, as lineages evolved to become polyplastidic, the emergence of stromules could represent an evolutionary adaptation to coordinate functions among multiple plastids within a single cell (Schattat *et al*., 2012b; Delfosse *et al*., 2018).

Plastids evolved from ancient bacteria, and their many bacterial-like characteristics suggest that their might use processes similar to phenomena such as quorum sensing in bacteria. If substantiated, this mechanism could be critical for synchronizing stress responses, regulating plastid size and number, and coordinating plastid division. However, it is important to note that this hypothesis remains entirely conjectural and lacks empirical evidence.

The idea that stromules contribute to plastid signaling or synchronization, akin to bacterial communication systems, is intriguing but requires substantial experimental validation. Future studies should investigate whether stromules enable functional connectivity between plastids and explore their potential roles in stress response and cellular coordination. Understanding these mechanisms could provide valuable insights into the evolution and functionality of plastid networks in plant cells. In this context, it is particularly noteworthy that plastids retain several bacterial-like features, including their mode of division. Plastids divide by binary fission, a process strikingly similar to that used by free-living bacteria and mediated by homologous proteins such as FtsZ, MinD, and ARC6 (Vitha *et al.*, 2003; Hirakawa and Ishida, 2015; Irieda and Shiomi, 2017). This evolutionary conservation highlights the deep prokaryotic roots of plastids and suggests that their modern dynamics—including potential inter-organelle communication via stromules—might have evolved from ancient mechanisms of cellular coordination. Exploring how these conserved division systems interact with plastid extensions such as stromules could shed light on whether such structures play roles in synchronizing plastid proliferation, function, or signaling during development and environmental adaptation.

### Plastid–ER interactions

Close interactions between the ER and plastids have been extensively studied (Figs 5, 6; Table 1), but their precise nature, including the presence of distinct and specialized contact domains, remains unclear (Breuers *et al*., 2012; Wang and Benning, 2012; Poulsen *et al*., 2015; Botella *et al*., 2016; Mathur *et al*., 2023). For instance, the observation of leucoplasts synthesizing precursors for secreted compounds, coupled with their close physical association with the ER and the presence of shared or sequential steps in biosynthetic pathways across these two organelles, provides strong evidence for a functional interaction between leucoplasts and the ER in plant secretion processes. Structures such as stromules, which have been observed to associate with the ER (Barton *et al.*, 2018), might play a role in these connections. However, the nature and identity of specialized biomolecules essential for stabilizing the ER–plastid interface are not yet fully understood (Mehrshahi *et al.*, 2014; Stefano *et al.*, 2014; Wang *et al.*, 2023).

A close association between the ER and plastids has been proposed, and recent evidence highlights the role of the ER in mediating interactions between organelles (Andersson *et al.*, 2007; Mathur *et al.*, 2022). Using targeted multicolored fluorescent fusion proteins, researchers have tracked the dynamic spatiotemporal interactions among plastids, mitochondria, peroxisomes, and the ER in living plant cells. These studies have revealed that while smaller organelles, such as plastids, mitochondria, and peroxisomes, do not form direct interactions with each other under normal growth conditions, they all maintain regular contact with the ER. Under stress, such as high light intensity, these associations become more pronounced, suggesting that the ER acts as a central mediator, coordinating organelle interactions and stress responses (G. Stefano *et al*., 2015; Mathur *et al*., 2022).

Light microscopy using optical tweezers has demonstrated that the ER network follows chloroplast movement, providing strong evidence for the close association between the ER and plastids (Andersson *et al.*, 2007). The ER network is hypothesized to act as a hub for assembling MCSs between organelles (Bian *et al.*, 2023; Wang *et al.*, 2023). Preliminary research identified potential proteins involved in these MCSs, such as VAP27, which interacts with chloroplast envelope-localized viral proteins during infection (Wei *et al.*, 2013). Further evidence from transgenic plants expressing fluorescent fusion proteins targeted to both the ER and plastid stroma has indicated that transient MCSs form between these organelles (Mathur *et al.*, 2023). However, more research is needed to identify and characterize the molecular machinery responsible for forming and maintaining these contact sites and to define its physiological role.

Plastids and the ER share biosynthetic pathways, particularly for fatty acid and lipid synthesis, necessitating close collaboration between these organelles (Wang and Benning, 2012; Block and Jouhet, 2015). Lipid trafficking between plastids and other endomembrane compartments appears to be independent of vesicular transport in many plant species (Villarejo *et al.*, 2005; Jarvis and López-Juez, 2013; Hurlock *et al.*, 2014). Instead, it probably involves direct contact sites such as plastid-associated membranes, identified using freeze-fracture scanning and transmission electron microscopy (Mehrshahi *et al*., 2013, 2014; Block and Jouhet, 2015). These structures are thought to facilitate bidirectional transport of non-polar molecules between the ER and plastids.

Supporting evidence comes from biochemical studies showing that isolated chloroplasts retain portions of the ER membrane and from transorganellar complementation experiments in which plastid-targeted enzymes retargeted to the ER restore the wild-type phenotype in mutants with compromised plastid metabolism (Andersson *et al.*, 2007; Mehrshahi *et al.*, 2013). While these findings underscore the functional significance of ER–plastid contact sites, particularly in lipid biosynthesis and metabolic coordination, the underlying mechanisms enabling metabolite exchange—such as membrane hemifusion—remain hypothetical. Despite initial proposals in the original studies (Mehrshahi *et al.*, 2013), direct evidence for membrane continuity or fusion between organelles is still lacking. Continued investigation is therefore essential to clarify how such close physical associations translate into functional exchange.

Recent research has also shed light on the identity of key molecules involved in the ER–chloroplast MCSs. Specifically, the VAP27 proteins in the ER membrane and the lipid-binding protein ORP2A have been shown to form a functional complex at the ER–chloroplast contact sites (Renna *et al.*, 2024). This work has also shown that VAP27 interacts with the chloroplast OEM, binding to ORP2A, which in turn interacts with the lipid monogalactosyldiacylglycerol (MGDG) in the chloroplast outer-envelope membrane. Loss of the VAP27–ORP2A complex results in subtle changes to the acyl composition of MGDG and phospholipid phosphatidylglycerols, as well as alterations in sterol levels in the chloroplasts. These findings suggest the innovative hypothesis that the VAP27–ORP2A complex is critical for maintaining lipid homeostasis at these MCSs and support the role of the ER in mediating lipid trafficking and metabolic coordination between the ER and plastids. In contrast, *Paulinella chromatophora*, a model for a more recent and independent primary endosymbiosis, shows stronger evidence for ER-mediated transport into its photosynthetic organelles (chromatophores), including vesicle-based pathways and possible MCSs (Nowack and Grossman, 2012). This clearer linkage between host endomembrane systems and organelle protein-targeting in *Paulinella* highlights both parallels and divergences in how primary endosymbiotic relationships have evolved, offering insights into early organelle integration pathways that might have been transient or less easily preserved in the Archaeplastida lineage (Nowack and Grossman, 2012).

Although stromules are often observed in close association with the ER and other organelles (Schattat and Klosgen, 2011; Schattat *et al.*, 2011; Barton *et al.*, 2018), whether they directly facilitate lipid exchange between plastids and the ER remains unclear. Their potential involvement in ER–plastid interactions is an important area for future research. Other key questions include whether stromules form specific contact sites with the ER and whether they contribute to the bidirectional transport of lipids or other molecules. Another critical area of investigation is the role of specialized biomolecules in stabilizing the ER–plastid interface (Mehrshahi *et al.*, 2014; Stefano *et al.*, 2014).

### Plastid–Golgi/*trans*-Golgi network interactions

Plastids are closely associated with Golgi bodies (Figs 5, 6), although this association is shorter in duration compared with plastid–peroxisome interactions, being ∼3.5 times shorter (Gao *et al.*, 2016).

Some nuclear-encoded proteins destined for plastids utilize unconventional translocation pathways, by-passing the TOC–TIC machinery (Villarejo *et al.*, 2005; Richardson and Schnell, 2020). These proteins appear to travel from the ER to the Golgi via vesicles, where they undergo glycosylation before being delivered to the plastid envelope (Fig. 3B). Examples of such proteins include α-amylase I-1 (AmyI-1) (Kitajima *et al.*, 2009), α-type carbonic anhydrase 1 (CAH1) (Villarejo *et al.*, 2005), p43 (Gaikwad *et al.*, 1999), manganese superoxide dismutase 1 (MSD1) (Shiraya *et al.*, 2015), and nucleotide pyrophosphatase/phosphodiesterase proteins (NPP)1, NPP2, and NPP6 (Nanjo *et al.*, 2006; Kaneko *et al.*, 2016). This suggests that some proteins follow a secretory pathway, entering the ER, passing through the Golgi, and ultimately reaching plastids (Kitajima *et al.*, 2009).

*trans*-Golgi compartments play a role in protein targeting and trafficking from the Golgi to plastids (Baslam *et al.*, 2016). These compartments, a collection of Golgi-associated vesicles, direct proteins to various destinations, including plastids. For instance, NPPs are transported to *trans*-Golgi compartments where they undergo glycosylation modifications before being delivered to plastids via vesicles (Baslam *et al.*, 2016). Although the precise targeting mechanisms are not fully understood, it is hypothesized that *trans*-Golgi compartments might house receptors that recognize specific sequences in NPPs, mediating their transport to plastids.

These findings not only emphasize the critical role of *trans*-Golgi compartments in plastid targeting but also suggest potential regulatory mechanisms governing this process. Further research could uncover additional proteins trafficked through this pathway and clarify how these interactions are regulated. While the exact mechanism remains uncertain, it is plausible that contact sites between plastids and the Golgi are involved in facilitating this transport.

### Plastid–endosome interactions

Chloroplasts and endosomes have been shown to maintain close associations (Wu *et al.*, 2020) (Fig. 5), raising the possibility of specialized contact sites between these organelles. This interaction is reminiscent of the established mitochondria–endosome connections in eukaryotic cells, which play significant roles in mitochondrial quality control, ion and lipid transfer, and release of extracellular vesicles (Todkar *et al.*, 2019).

Plastids and mitochondria share a common evolutionary origin, having emerged from endosymbiotic bacteria that were engulfed by a eukaryotic ancestor (Snyder and Stefano, 2015; G. B. Stefano *et al.*, 2015). Insights from mitochondria–endosome studies might provide valuable clues about plastid–endosome interactions.

Evidence suggests that endosomes can take up proteins from plastids that are no longer needed, hinting at potential roles in cellular recycling or signaling (Spitzer *et al.*, 2015). However, plastid–endosome interactions are a relatively new field of study, and much remains unknown. Further research is needed to confirm the existence of contact sites between plastids and endosomes and to explore their functional significance, particularly in cell signaling and metabolic coordination.

### Plastid–peroxisome interactions

Plastids, particularly chloroplasts, share common metabolic pathways with peroxisomes, such as their involvement in photorespiration (Oikawa *et al.*, 2015, 2019). Electron and confocal microscopy of leaf and cotyledon cells has revealed close spatial associations between chloroplasts and peroxisomes (Figs 5, 6). In cotyledon cells, inactivating the RING finger domain of peroxin 10 (PEX10) disrupts the physical interaction between these two organelles, impairing metabolite exchange (Schumann *et al*., 2007) (Table 1). However, it remains unclear whether the resulting phenotypes are due to the physical uncoupling of plastids and peroxisomes or to a broader loss of peroxisome functionality that indirectly impacts plastid metabolism.

Despite evidence of close proximity, a study has found no direct physical interactions between plastids and peroxisomes under normal conditions (Mathur *et al.*, 2022). Instead, frequent contact with the ER is observed, and ER membranes are consistently present between these organelles, even when they appear clustered. This raises the possibility that the ER acts as a mediator of organelle interactions, facilitated by its association with cytoskeletal elements such as microtubules and actin filaments (Ueda *et al.*, 2010; Hamada *et al.*, 2014). The cytoskeleton provides structural support and tracks for molecular motors, such as dynein and kinesin, that transport organelles throughout the cell, potentially positioning the ER between other organelles.

The limitations of confocal microscopy, which lacks the resolution to detect the 10–80 nm spatial range of close contacts (Wang *et al.*, 2023), might also obscure direct organelle interactions. Higher-resolution techniques are essential for studying these interactions in greater detail. Recent advances in super-resolution microscopy offer promising alternatives. Techniques such as stochastic optical reconstruction microscopy (STORM) and stimulated emission depletion (STED) microscopy can achieve resolutions as fine as 20–50 nm, enabling more accurate visualization of close-range organelle interactions (Jans *et al.*, 2013; Bharadwaj *et al.*, 2024; Zanellati *et al.*, 2024). These approaches hold considerable potential for elucidating whether plastids and peroxisomes engage in direct physical contact or are consistently separated by intervening ER membranes. While the extensive presence of the ER in the cytosol might facilitate connectivity among organelles, its prominent association with the cytoskeleton might explain its frequent observation between organelles.

Biochemical experiments have also provided evidence of plastid–peroxisome interactions. For instance, sucrose-gradient centrifugation of spinach leaf extracts has demonstrated *in vitro* interactions between these organelles (Schnarrenberger and Burkhard, 1977). Furthermore, microscopy of Arabidopsis mutants defective in chloroplast photo-relocation have revealed that while other organelles lose coordinated movement with chloroplasts, peroxisomes remain closely associated (Oikawa *et al.*, 2003; Shai *et al.*, 2016). However, these associations do not necessarily indicate direct membrane contact, and alternative explanations should be considered, such as shared anchoring mechanisms or coordinated responses to cytoskeletal dynamics.

A notable technique used to study plastid–peroxisome interactions is optical tweezers, which employ light beams to manipulate microscopic objects such as organelles. This approach has demonstrated that peroxules—protrusions from peroxisomes—physically bridge peroxisomes and chloroplasts. In dynamic cellular environments, peroxules play essential roles in facilitating interactions for key biochemical processes, such as fatty acid mobilization, photorespiration, redox metabolism, and hormone biosynthesis (Sinclair *et al.*, 2009; Gao *et al.*, 2016).

Although there is extensive evidence for plastid–peroxisome interactions, the biomolecules mediating these contacts in plants remain unidentified. In contrast, studies in non-plant systems have uncovered numerous molecular machineries involved in peroxisome–endomembrane interactions. Unlike plastids and mitochondria, which trace their origins to endosymbiotic bacteria, peroxisomes are thought to derive from the endomembrane system. According to Tabak *et al*. (2013), they form *de novo* from the ER through vesicle budding and maturation. This endogenous origin might account for their close association with the ER and spatial interactions with other organelles. Thus, a more critical appraisal of plastid–peroxisome functional coupling in plants is needed. Specifically, future studies should distinguish between defects arising from disrupted physical contact versus broader metabolic dysfunction due to impaired peroxisomal activity. In the absence of clear mechanistic insight, interpretations of mutant phenotypes might inadvertently conflate correlation with causation, leading to potentially misleading conclusions. In addition to plastid–organelle interactions, other inter-organellar interactions with the secretory membranes that do not involve plastids occur in eukaryotic cells. For completeness, here we describe the most-characterized to date.

Vesicles have also been implicated in transporting biomolecules to peroxisomes in both mammalian and yeast cells (Neuspiel *et al.*, 2008; Lam *et al.*, 2011).

In plant systems, the molecular machinery mediating plastid–peroxisome interactions remains largely unexplored. While it is tempting to hypothesize that plants might share similar mechanisms with non-plant systems, such as vesicle-mediated transport and protein-mediated contact sites, direct evidence is lacking (Pan *et al.*, 2019). Further research is needed to identify the specific players involved in plastid–peroxisome contact sites and to elucidate their roles in processes such as metabolite exchange and signaling.

### Plastid–mitochondria interactions

Microscopy studies have revealed close associations between plastids and mitochondria (Figs 5, 6), which is not surprising given their interdependence in energy metabolism. Plastids provide substrates for mitochondrial respiration, while mitochondria supply essential intermediates for photosynthetic carbon assimilation. These interfaces probably play a key role in facilitating fast communication and efficient biomolecule exchange between the two organelles.

Confocal microscopy studies have shown clusters of mitochondria around plastid stromules, suggesting potential contact sites that promote communication and biomolecule transfer (Kwok and Hanson, 2004c). This close relationship has been further examined under stress conditions, such as phosphate starvation, where the number of contact sites between plastids and mitochondria significantly increases (Jouhet *et al.*, 2004; Michaud *et al.*, 2016). This dynamic response indicates that plastid–mitochondria interactions are modulated by environmental and metabolic cues.

Research in Arabidopsis has identified the mitochondrial transmembrane lipoprotein (MTL) complex, which facilitates galactoglycerolipid transfer between plastids and mitochondria (Michaud, 2018). The MTL complex includes proteins from both the mitochondrial membrane and the plastid envelope, highlighting its role as a specialized structure at the plastid–mitochondria junction (Michaud *et al.*, 2016). This complex is one of the few directly identified contact sites involved in a specific type of transfer: that of galactoglycerolipids. While progress has been made (and more mechanisms are being discovered), the full breadth of molecular components facilitating direct inter-organellar communication between plastids and mitochondria remains an active area of research with many unknowns. Studies under phosphate starvation conditions offer a promising model for investigating these lipid transfer processes further (Li-Beisson *et al.*, 2017).

### Plastid–plasma membrane interactions

Plastids are dynamic organelles whose movement is cytoskeleton-dependent and influenced by environmental factors, such as light intensity (Whippo *et al.*, 2011; Sakai and Takagi, 2017). This mobility allows them to form temporary associations with the plasma membrane (PM) for specific functions, such as the export of digalactosyldiacylglycerol (DGDG), a galactolipid synthesized in the chloroplast and delivered to the PM (Mueller-Schuessele and Michaud, 2018). A close association between plastids and the PM could facilitate the efficient transfer of DGDG to maintain membrane composition and functionality (Jouhet *et al.*, 2004).

Experimental evidence suggests that plastid–PM interactions are mediated by a chloroplast outer-envelope protein CHUP1, which uses its coiled-coil domain to establish connections between the plastid and the PM (Oikawa *et al*., 2008). Despite advances in confocal microscopy and TEM revealing the intimate nature of plastid–PM interactions, it remains unclear whether they involve membrane contact sites or vesicular transport for DGDG delivery (Michaud and Jouhet, 2019).

### Plastid–vacuole interactions

The close associations between plastids and vacuoles appear to be tissue-specific and might reflect the unique metabolic demands of different cell types. For example, in nectary tissue during periods of active nectar secretion, amyloplasts become closely associated with vacuoles (Figs 5, 6; Table 1). These associations facilitate the transfer of starch grains from plastids to the vacuole sap, where the starch is depolymerized and utilized (Pacini *et al.*, 2003; Gaffal *et al.*, 2007). These interactions probably play a key role in supporting the high energy demands of nectar production.

The specific proteins and mechanisms facilitating direct, non-degradative material exchange between plastids and vacuoles through membrane evaginations or small openings remain unknown. Further research is needed to explore whether these contact sites are specific to nectary tissue or represent a broader mechanism for plastid–vacuole communication in plants.

In addition to plastid-specific interactions, other vital inter-organellar communications occur within eukaryotic cells, particularly involving the secretory membranes. For completeness, below we describe the most-characterized to date, including well-established interactions of peroxisomes with organelles such as the endoplasmic reticulum and mitochondria. Given the ubiquitous role of peroxisomes in metabolism and their intimate association with both plastids and mitochondria in plants (e.g. in photorespiration), it is highly probable that they also engage in direct contact with plastids in plant cells, although specific molecular tethers for this interaction remain an active area of research.

### Peroxisome–ER interactions

The sites where interactions take place are critical for lipid trafficking and peroxisome membrane expansion, and they have been mainly explored in mammals. The acyl-CoA binding domain-containing proteins ACBD4 and ACBD5 on the peroxisomal membrane tether to VAMP-associated protein (VAP)A and VAPB on the ER, forming a stable complex mediating lipid exchange and organelle positioning. These proteins bind directly to fatty acids and acyl-CoA esters, facilitating the transfer of lipids between compartments and highlighting the metabolic integration between peroxisomes and the ER (Castro *et al*., 2018; Islinger *et al*., 2018).

### Peroxisome–lysosome interactions

Although less well characterized, these interaction sites appear to play roles in lipid metabolism and signal transduction. Synaptotagmin 7 (SYT7), a Ca^2+^-sensing protein, and the signaling lipid phosphatidylinositol 4,5-bisphosphate [PI(4,5)P_2_] are implicated in forming and regulating these interactions in mammalian cells. SYT7 might mediate Ca^2+^-dependent tethering, while PI(4,5)P_2_ acts as a membrane signal for initiating contact and possibly lipid remodeling or degradation processes (Castro *et al*., 2018).

### Peroxisome–mitochondrion interactions

In yeast, the peroxisomal proteins Pex11 and Pex34 form contact sites with mitochondria by interacting with the outer-membrane proteins Fuzzy Onions homolog 1 (Fzo1) and Mitochondrial Distribution and Morphology protein 34 (Mdm34). Fzo1 aids mitochondrial fusion, while Mdm34, part of the Endoplasmic Reticulum and Mitochondria Encounter Structures (ERMES) complex, mediates membrane tethering and lipid exchange (Castro *et al.*, 2018).

## Conclusions

Significant progress has been made in understanding plastid origins, genome evolution, and protein import machinery, shedding light on the intricate integration and coordination of plastids within plant cells. The endosymbiotic theory has provided a foundational framework to explore the evolutionary relationship between cyanobacteria and plastids. Key similarities between them including DNA sequence homology, shared protein machinery, and transmembrane-barrel proteins, offer compelling evidence supporting this theory. Through endosymbiosis, plastids have undergone extensive genome evolution, with much of their genetic material being transferred and integrated into the host-cell nucleus. The presence of a reduced plastome, which retains essential genes for ribosomal components, tRNA, and organellar proteins, underscores the dependence of plastids on the nucleus for biogenesis and function.

Advancements in understanding the plastid protein import machinery, particularly the TOC–TIC translocon complex, have clarified the precise mechanisms by which proteins are transported into plastids. This intricate process, involving receptor proteins, channel-forming proteins, and other translocon components, ensures the accurate targeting of proteins to plastid sub-compartments. This specificity is critical for the proper folding and assembly of proteins into functional forms, enabling the diverse roles of plastids in cellular metabolism. The identification and characterization of these import components have deepened our understanding of plastid protein import, yet significant knowledge gaps remain.

One key area requiring further investigation is the communication between plastids and other cellular compartments. Evidence of inter-organelle membrane contact sites suggests that plastids coordinate activities and exchange metabolites and information with other organelles. However, the exact mechanisms and regulatory factors governing these interactions remain poorly understood. Additional research is needed to elucidate the dynamics and functional significance of these inter-organelle connections.

Another unresolved topic is the import of specific protein classes, notably glycosylated proteins, into plastids. While some studies suggest alternative pathways that by-pass the canonical TOC–TIC machinery for these proteins, the precise mechanisms and regulatory factors facilitating such processes are not yet fully characterized. Understanding these pathways and their molecular players will provide valuable insights into the metabolic cooperation and functional diversity of plastids within plant cells.

Thus, while our understanding of plastid origins, genome evolution, and protein import machinery has advanced considerably, further research is essential to uncover the mechanisms underlying plastid communication and specialized protein import, and we conclude with some ‘open questions’ below. Continued exploration of these areas will contribute to a more comprehensive understanding of plastid functions and their pivotal roles in metabolic cooperation within plant cells. Such knowledge holds significant potential for applications in plant biotechnology, agriculture, and the broader study of eukaryotic cell evolution.

### Open questions

Despite significant advances in understanding plastid evolution, organelle communication, protein import, and differentiation, numerous questions remain unanswered. For instance, the mechanisms controlling plastid differentiation are still poorly understood, especially when considering the remarkable diversity of plastid forms across different plant species and between parasitic and non-parasitic species (Krause, 2008; Wicke and Naumann, 2018). Indeed, the field is ripe with open questions related to several key aspects, which are outlined below.

#### Plastid protein import

The identification of uncharacterized import machinery outside the TOC–TIC complex represents a major topic for further investigation. The prevailing view is that protein import into chloroplasts occurs via the TOC–TIC complex anchored to their outer- and inner-envelope membranes, respectively. However, evidence suggests that some glycosylated proteins reach plastids after passing through other organelles, such as the Golgi apparatus (Fig. 1). This raises the possibility of alternative pathways for protein import into plastids. To date, it has not been demonstrated that the TOC–TIC machinery can transport glycosylated proteins, supporting the hypothesis of a direct transport route from the Golgi to plastids. The molecular mechanisms underlying this potential pathway are unknown, and further research is needed to determine whether N-glycosylated proteins are imported through an entirely distinct mechanism or via a yet-to-be-characterized modification of the TOC–TIC system.

#### Plastid–plastid communication

Another intriguing question is whether the observed connections between plastids, often mediated by stromules, play a role in communication, biomolecule exchange, or both. Several studies have reported close associations between plastids via stromules (Breuers *et al.*, 2012; Hanson and Sattarzadeh, 2013; Hanson and Hines, 2018). One hypothesis is that this system could function as a signal transduction mechanism, where a signal captured by one plastid is transmitted to others in a chain reaction, potentially enabling a coordinated and efficient cellular response. Experimental evidence is needed to validate or refute this hypothesis.

#### Plastid–nucleus interactions

The role of close associations between plastids and the nucleus in communication remains unresolved. Plastids continuously communicate with the nucleus, often sending retrograde signals to regulate their physiological state in response to environmental changes. Observations of plastid stromules interacting with the nucleus suggest that these structures might act as channels for communication, analogous to tunneling nanotubes or cytonemes (Köhler and Hanson, 2000; López-Juez, 2007; Caplan *et al*., 2015; Irieda and Takano, 2021). Stromules might facilitate the transfer of signaling molecules, such as proteins, or deliver retrograde signals while maintaining their informational integrity. Further experimental evidence is required to confirm whether stromules serve this role, but this represents a promising avenue for future research.

#### Plastid–endomembrane contact sites

The existence of other MCSs involving plastids is another area for exploration. For example, plastid–endosome contact sites have not yet been reported. However, drawing parallels with mitochondria—which also originated from prokaryotic organisms—suggests that plastids might engage in similar interactions. In mammalian cells, mitochondria are known to interact with endosomes physically and functionally, playing roles in processes such as vesicle trafficking and ion transfer (Todkar *et al.*, 2019). It is reasonable to hypothesize that plastids might form analogous contact sites with endosomes, although further research is needed to test this.

#### Tissue-specific and developmental variability

Another unresolved question concerns whether plastid interactions vary depending on the developmental stage of the organism or the tissue type involved. Plastid functions and associations are known to differ between tissue types, but the extent to which these interactions are influenced by developmental or environmental factors remains unclear.

## Data Availability

This manuscript is a review article and does not report new datasets. The images presented were generated in our laboratories for illustrative purposes and are based on previously published evidence, which is duly cited in the text. No additional datasets were created or analyzed in the preparation of this work.
